# The prevalence of internalized stigma and its association with HIV viral suppression among fully disclosed adolescents and young adults living with HIV (AYLHIV) receiving HIV care in an HIV clinic in Plateau State, Nigeria

**DOI:** 10.1371/journal.pone.0303360

**Published:** 2024-05-13

**Authors:** Emmanuel O. Osayi, Oluseye Ajayi, John Onyeji, Mercy Isichei, Atiene S. Sagay, Albert Anderson

**Affiliations:** 1 Directorate of Clinical Services, APIN Public Health Initiatives Jos, Plateau, Nigeria; 2 Directorate of Prevention, APIN Public Health Initiatives FCT-Abuja, Abuja, Nigeria; 3 Department of Obstetrics and Gynaecology, Bingham University Teaching Hospital, Jos, Plateau, Nigeria; 4 Department of Surgery, Jos University Teaching Hospital, Jos, Plateau, Nigeria; 5 Department of Obstetrics and Gynaecology, Jos University Teaching Hospital, Jos, Plateau, Nigeria; 6 Grady Health System, Emory School of Medicine, Atlanta, GA, United States of America; University of Manitoba Faculty of Health Sciences, NIGERIA

## Abstract

Internalized stigma, a condition characterized by negative self-stereotyping and social alienation, recently impacted the adolescents and young adults living with HIV (AYLHIV) epidemic curve and treatment adherence. While prior research has focused on the impact of internalized stigma among adults living with HIV, few studies focused on this AYLHIV. The study aims to determine internalized stigma proportion and its relationship to HIV viral suppression in AYLHIV. A cross-sectional study involved 93 fully disclosed AYLHIV receiving HIV care in Faith Alive Foundation in Jos North, Plateau State, from January to March 2023. Internalized stigma was measured using the adapted Berger HIV Stigma Scale under the domains personalized stigma (18 item questions) and negative self-image subscales (13 item questions), measured on a 4-scale of strongly disagree (1), disagree (2), agree (3), and strongly agree (4). Scores summed up to give the domain composite score with a maximum obtainable score of 72 for personalized stigma and 52 for negative self-image. A total of 93 respondents, female-63 (68%) and male-30 (32%), were involved in the study and their mean age at full disclosure was 15.7 ± 2.8 years. During the study their mean age was 19.5 ± 5.4 years, with 62% (58) ages 10–19 years and 38% (35) ages 20–26 years. Furthermore, 70% of the participants had secondary educational status, 77% had viral load results <1000 copies/ml), and 57% were on ART for up to 6 years. The average scores for personalized and negative self-image were 36.3 and 28.9, with 53% (49/93) and 52% (48/93) scoring higher than the average respectively. Further subclassification of the participants by the presence of internalized stigma domains reported 62% (58/93) with both domains, 20% (19/93) with at least one domain, and 38% (35/93) with none of the domains. Negative self-image stigma was reported more among participants 10–19 years (63%), male (31%), of secondary educational level (71%), virally unsuppressed (23%), and ≤ 6 years on ART (42%). On the other hand, personalized stigma was more among the female participants (73%), ages 20–29 years (41%), educational level (6% and 27% had primary and tertiary level of education respectively), virally suppressed (80%), and up to 6 years on ART (63%). The correlation between the internalized stigma domains and suppressed viral load using a binary multivariate regression method at 95% CI and a p-value of 0.05 was not statistically significant with personalized stigma (*p* = 0.73) and negative self-image (*p* = 0.92). The adjusted odds ratio of having internalized stigma among the virally suppressed were personalized stigmas [OR; 1.21, 95% CI; 0.42–3.47] and that of negative self-image [OR; 1.06, 95% CI; 0.38–2.95]. This study showed a high proportion of internalized stigma among females, ages 10–19 years, and virally suppressed with more odds for personalized stigma domain. However, the study reported no statistically significant association between internalized stigma domains and viral suppression.

## Introduction

According to UNAIDS, adolescents aged 10–19 and youths aged 15–24 [1] represent a growing population of people living with HIV. In 2019, an estimated 1.7 million and 3.4 million adolescents and youths were living with HIV [2], and by 2020, the estimated number of adolescents living with HIV (ALHIV) grew by five percent [3]. Similarly, in Nigeria, approximately 3.5 percent (66,500) of the 1.9 million people living with HIV were adolescents, the highest among countries in West and Central Africa, and by 2020, the number of ALHIV in Nigeria increased to 97,000 [4]. Internalized stigma, with its complex psychosocial challenges, has been on the rise among the group ages 10–19 years and 20–24 years and could be a contributor to the HIV epidemic [5].

According to the HIV-related stigma framework, internalized stigma, also known as self-stigma, occurs when adolescents absorb negative messages and stereotypes about HIV (negative self-image stigma), come to personalize them, and apply them (personalized stigma) [5]. The presence of either both negative self-image stigma and personalized stigma or one of it could lead to the manifestation of existing or new mental and psychosocial symptoms that may affect the patient’s adherence to antiretroviral therapy and the treatment outcome [6]. Studies conducted in the United States of America showed that a significant number of people living with HIV experienced internalized stigma, especially among newly identified HIV-positive clients [7,8]. Again, a study conducted in Kenya showed that internalized stigma could present in adolescents living with HIV who had self-disclosed [9]. The latter could be due to HIV still being highly stigmatized in sub-Saharan African countries, including Nigeria, and the people living with HIV may come to accept these perceptions as valid and thereby develop internalized stigma [10]. To better manage the burden of this type of stigma among the adolescents living with HIV in the country, the Federal government of Nigeria adopted an HIV disclosure strategy [10].

The HIV disclosure strategy is an ongoing process in which a newly identified HIV-positive child/adolescent is informed about their HIV status and may willingly share it with a family member, friend, or significant others [8]. HIV disclosure is an age-appropriate support offered to children/adolescents to feel socially acceptable to themselves and society. HIV disclosure is in phases, the minimum being a partial disclosure at ages 5–9 years, full disclosure at ages 10–12 years, and psychosocial support to transition to other relevant programs at ages 13–19 years [10]. However, the transitioning process is flexible and client-centered and sometimes extends into the young adulthood age 26 years [1,10]. Hence, the reason our study age group extends to 26 years.

Studies have shown that a significant number of this population have attained the phase of full disclosure. A study conducted in Uganda showed that up to 30 percent of HIV-infected children aged 5–17 years achieved full HIV status disclosure [11]. Similarly, a study in Zimbabwe reported that 27 percent of HIV-positive children ages 9 to 15 years received full disclosure [12]. A cross-sectional study carried out in Abuja, Northern part of Nigeria, also recorded an overall disclosure rate of 47.2 percent among HIV-positive children aged 5–16 years on antiretroviral therapy for at least one (1) year, of which 24.5 percent had full disclosure [13].

HIV-positive serostatus disclosure has the advantage of empowering the clients to be pro-social, enhancing their access to social support, and reducing HIV transmission risks [14–16]. Although there are studies on disclosure, there are limited studies on the rate of internalized stigma among fully disclosed AYLHIV and its association with their viral load suppression.

This study sought to evaluate the rate of internalized stigma and its association with viral load suppression among AYLHIV ages 10–26 in Faith Alive Foundation, Jos North, Plateau State, Nigeria.

## Methods

### Study site

The study was conducted in Faith Alive Foundation (FAF) hospital located in Jos North local government area of Plateau State, Nigeria. The facility is high volume, with over 5000 people of different age groups living with HIV, and has a well-trained multidisciplinary team of health care workers that provide integrated comprehensive HIV care, prevention, and treatment services. Within the facility is an adolescent-friendly clinic with trained nurses that provide adolescent-friendly health services such as age-appropriate HIV status disclosure and psycho-social support for transitioning to adult ART clinics in line with the national HIV guideline [10].

### Study population, inclusion, and exclusion criteria

The study population is a cross-section of AYLHIV between the ages 10–26 years that have attained full disclosure of their HIV status and are in the process of transitioning to the adult ART clinic, accessing HIV care, prevention, and treatment at the study facility. Other inclusion criteria include being active on ART drug refill, greater than six months on ART, and having at least one viral load result post full HIV status disclosure. Clients with no date of achieving full disclosure, less than six months on ART, and without viral load results post-HIV disclosure were excluded from the study.

### Ethical consideration and consents

Before the participants’ selection, the study proposal assigned number FAFEC/08/34/25 was reviewed and approved by the Faith Alive Foundation Health Research ethics committee. Verbal and written consents were obtained individually from the clients reached and accepted to participate in the study, with the option to opt-out during the recruitment phase.

### Study sampling method and Recruitment process

The study sampling method was purposive with clients selected from the facility’s adolescent-friendly clinic register and the Nigerian Management Record System (NMRS) based on the inclusion criteria. The recruitment process was in three months, from the 1st of January to the 31st of March 2023. Selected clients were reached using their ART refill appointment dates and the facility’s quarterly adolescent and young adults club meeting, compulsory for all the target age groups accessing ART in FAF hospital.

The facility had a total of 117 AYLHIV registered in their facility’s adolescent-friendly clinic and their Nigerian Management Record System (NMRS) as at the time of the study. Following sorting, the study selected 93 clients based on its inclusion criteria. The remaining 24 clients were excluded from the study due to missing data on HIV disclosure date and viral load result (6), more than six months on ART but no post-disclosure VL result (11) and less than 6 months on ART and no post-disclosure VL result (7).

### Data collection and analysis

Data on internalized stigma was collected by the trained facility adolescent-friendly clinic nurse using the adapted Berger HIV Stigma questionnaire and measured under two domains: personalized stigma (18 item questions) and negative self-image subscales (13 item questions) [17–19]. Negative self-image stigma was described as when a client absorbs negative messages and stereotypes about HIV, while personalized stigma was described as when the client goes ahead and personalize them / or apply them to oneself [5].

The study measured each question on the adapted Berger HIV stigma questionnaire with a Likert 4-scale: strongly disagree (1), disagree (2), agree (3), and strongly agree (4), and the scores summed up to give the domain composite score with maximum obtainable scores of 72 and 52 for personalized stigma and negative self-image respectively [17]. Each domain was dichotomized into presence or absence using the average score. Scores higher than the average indicated the presence of the variable of interest, while scores lower than the average indicated its absence.

The variable viral load result was categorized into virally suppressed (VL <1000 copies/ml) and virally unsuppressed (VL ≥ 1000 copies/ml) [10] and measured the clients’ treatment outcomes. Other variables collected during the study were participants’ duration on ART and their sociodemographic status. The study null hypothesis (ho) was no association between the variable of interest and the outcome variable, while the alternative hypothesis (hA) is that there is an association between the variable of interest and the outcome variable.

Data analysis involved the use of a spreadsheet and SPSS version 27. Frequencies, proportions, and the association between internalized stigma and viral load suppression were measured using a chi-square test and binary multivariate regression analysis to control for confounders. The odds ratio (*OD*) was used to explore the risk estimate of internalized stigma among the participants virally suppressed at alpha (α) 0.05 and 95% confidence interval (Cl).

## Results

A total of 93 respondents, female-63 (68%) and male-30 (32%), were involved in the study and their mean age at full disclosure was 15.7 ± 2.8 years. During the study their mean age was 19.5 ± 5.4 years, with 62% (58) ages 10–19 years and 38% (35) ages 20–26 years. Furthermore, 70% (65/93) of the participants had secondary educational level, 77% (72/93) had viral load result less than 1000 copies/ml, and 78% (73/93) on ART up to 9 years. The socio-demographic characteristics of the participants is shown on Table 1 below.

**Table 1:** The socio-demographic characteristics of the participants.

Variables	*Frequency (n = 93)*	%
**Age**		
10–19 years	58	62%
20–26 years	35	38%
**Sex**		
Female	63	68%
Male	30	32%
**Educational levels**		
Primary	6	6%
Secondary	65	70%
Tertiary	22	24%
**Viral load results**		
<1000 copies / ml	72	77%
≥1000 copies per ml	21	23%
**Duration on ART in years**		
1–9 years	73	78%
10–19 years	20	22%

The average of the composite scores for the personalized and negative self-image stigma were at 36.3 and 28.9 respectively. The proportion of the participants that scored higher than the average for personalized and negative self-image stigma were 53% (49/93) and 52% (48/93) respectively.

The study showed an internalized stigma prevalence (which is the proportion of the participants that had either both domains of internalized or any one of the domains) of 62% (58/93). Among the participants that had internalized stigma, 42% (39/58) had both the internalized stigma domains, while 20% (19/93) had at least one of the domains. Table 2 below shows the prevalence of internalized stigma and its distribution across the variables by the domains.

**Table 2:** The prevalence of internalized stigma and its distribution across the variables by the domains (n = 93).

Variables	Presence of internalized Stigma		Patterns of Internalized Stigma		
	(No) Internalized Stigma	(Yes) Internalized Stigma	Both internalized stigma domains	Personalized stigma domain	Negative Self-Image stigma domain
**Total**	35 (38%)	58 (62%)	39 (42%)	10 (11%)	9 (10%)
**Age groups**					
10–19 years	22 (24%)	36 (39%)	23 (25%)	6 (6%)	7 (8%)
20–26 years	13 (14%)	22 (24%)	16 (17%)	4 (4%)	2 (2%)
**Sex**					
Female	23 (25%)	40 (43%)	29 (31%)	7 (8%)	5 (5%)
Male	12 (13%)	18 (19%)	10 (11%)	3 (3%)	4 (4%)
**Educational level**					
Primary	3 (3%)	3 (3%)	2 (2%)	1 (1%)	0 (0%)
Secondary	25 (27%)	40 (43%)	27 (29%)	6 (6%)	7 (8%)
Tertiary	7 (8%)	15 (16%)	10 (11%)	3 (3%)	2 (2%)
**Viral load results**					
< 1000 copies/ml	27 (29%)	45 (48%)	31 (33%)	8 (9%)	6 (6%)
> = 1000 copies/ml	8 (9%)	13 (14%)	8 (9%)	2 (2%)	3 (3%)
**Duration on ART**					
1–9 years	31 (33%)	45 (48%)	25 (27%)	9 (10%)	5 (5%)
10–19 years	4 (4%)	13 (14%)	7 (8%)	1 (1%)	4 (4%)

The internalized stigma prevalence ratio (PR) was 1.01 times more among the respondents virally suppressed and aged 20–26 years to the respondents virally unsuppressed and aged 10–19 years respectively (See Table 3 below).

**Table 3:** The measures of association between the variables and internalized stigma.

Variables	(No) Internalized Stigma	(Yes) Internalized Stigma	Total		χ2	df	*p*-value	Crude OR	95% CI	
				PR					Lower	Upper
**Age groups**					0.01	1	0.94	0.97	0.41	2.30
10–19 years	22	36	58	0.62						
20–26 years	13	22	35	0.63						
		** **		**1.01**						
**Sex**										
Female	23	40	63	0.63	0.11	1	0.74	1.16	0.47	2.83
Male	12	18	30	0.60						
				**1.06**						
**Educational level**					0.73	2	0.69			
Primary	3	3	6	1.66						
Secondary	25	40	65	2.65						
Tertiary	7	15	22	3.55						
**Viral load results**										
< 1000 copies/ml	27	45	72	0.63	0.00	1	0.96	0.97	0.36	2.65
> = 1000 copies/ml	8	13	21	0.62	0.00	1	0.96	1.03	0.38	2.79
				**1.01**						
**Duration on ART**					0.00	1	0.96	1.03	0.38	2.79
1–9 years	31	45	81	0.56						
10–19 years	4	13	17	0.76						
				**1.38**						

P-value < 0.05 is statistically significant; The interaction between viral load results and internalized is shaded in orange.

Further analysis using chi square bivariate regression indicated no statistically significant association between internalized stigma and all the variables at 95% CI and p value 0.05 (See Table 3 below).

The crude Odds ratio (OR) and the 95% CI difference was greater than one which resulted to the equal chances of an internalized stigma among the virally suppressed occurring or not occurring.

On adjusting for confounders using binary multivariate regression analysis, the results of the odds ratio and the 95% CI difference was also greater than one. The latter also showed that having internalized stigma among the participants virally suppressed at 95% CI was just as it not happening. There was also no association between internalized stigma and viral load suppression with the p value greater than 0.05 (See Table 4 below).

**Table 4:** Multivariate analysis: Adjusting for confounders in the association between Internalized stigma and Viral load Suppression.

Variables	(No) Internalized Stigma	(Yes) Internalized Stigma			95% C.I. for β			
			Beta	SE	LL	UL	β	p-value
**Age groups**								
10–19 years	22	36	0.13	0.50	0.43	3.07	1.14	0.79
20–26 years	13	22						
**Sex**								
Female	23	40	0.17	0.49	0.45	3.12	1.19	0.72
Male	12	18						
**Educational level**								0.67
Primary	3	3						
Secondary	25	40						
Tertiary	7	15						
**Viral load results**								
< 1000 copies/ml	27	45	-0.00	0.53	0.35	2.84	1.00	1.00
> = 1000 copies/ml	8	13	0.00	0.53	0.35	2.86	1.00	1.00
**Duration on ART**								
1–9 years	31	45	0.35	0.55	0.35	3.02	1.03	0.95
10–19 years	4	13						

Note

* Β is adjusted Odds ratio (OD) and p-value 0.05 is statistically significant; The interaction between viral load results and internalized is shaded in orange.

## Discussion

The study population was adolescents aged 10–19 years and young adults aged 20–26 years who had achieved full HIV disclosure, of school age, and on lifelong antiretroviral therapy. The implication of achieving full HIV disclosure is that they feel socially acceptable to themselves and the society and have less HIV-related stigma. However, the study findings revealed a substantial prevalence of internalized stigma, 62 percent (58/93) among the participants, with a majority (42%) exhibiting both domains of the internalized stigma.

The study further revealed that most of the participants with internalized stigma were of the female sexes (40/58). This could be interrelated to the study location, the northern Nigeria, where it is perceived that the success of a young woman is tied to a man [20]. Again, most African countries, including Nigeria, have in existence a patriarchal system and its negative impact on the social development of women, including promoting stigma and discrimination. This could heighten the fear of rejection, abandonment, or partner violence among the women and limit the persons they disclose their HIV status [21]. The other angle to the discussion is that girls are more likely to keep their HIV status a secret than the male sexes which could imply low level of comfort in communicating their HIV status to others [21].

Furthermore, the study findings showed that participants of secondary educational level and aged 10–19 years had a high proportion of internalized stigma (40/58) and (36/58) respectively. On the contrary, findings from available studies showed dropping out of school increases internal stigma among the AYLHIV [22]. The study finding could be attributed to the levels of social support from the participants’ schoolmates, teachers, and friends. Studies have shown that schools have the potential to implement HIV stigma reduction programs that could give the needed high level of social support for the overall well-being of the AYLHIV [21].

There was also an intriguing finding of a high level of viral load suppression 77% (45/58), among the respondents with internal stigma with no statistically significant association between the two variables at 95% CI and a p-value of 0.05%. On the contrary, findings of available studies showed that internal stigma is associated with a lower level of viral suppression [23–25]. A possible explanation could be self-efficacy, which refers to an individual’s belief in her capacity to execute behaviors necessary to produce specific performance attainments [26]. In the study by Bruffell in 2017, individuals with HIV could be labeled by the society due to their positive status but have variance in the extent they internalize the labelling and its subsequent effect on their treatment outcome [27].

## Study limitations

The study recorded limited access to the client’s information in the facility’s adolescent youth-friendly ART clinic register and Nigerian Management Record System (NMRS) leading to the exclusion of 24 clients. Further study is required to explore the existence and impact of stigma reduction programs in the schools of the participants of secondary school level and aged 10–19 years. The study also revealed a potential need for further study to understand the self -efficacy of the virally suppressed study participants with internal stigma.

## Conclusion

This study reported a high burden of internalized stigma among the female sexes, age 10–19, and of secondary educational level and lower levels of viral load suppression. The burden of internalized stigma was slightly more of a personalized stigma with a higher risk estimate than negative self-image stigma. Although the association between internalized stigma domains and viral load suppression is not statistically significant, there is a need for the integration of routine screening of AYLHIV for internalized stigma in our Adolescent-youth-friendly ART clinics.
